# Genome-Wide Protein Interaction Screens Reveal Functional Networks Involving Sm-Like Proteins

**DOI:** 10.1002/1097-0061(20000630)17:2<95::AID-YEA16>3.0.CO;2-H

**Published:** 2000

**Authors:** Micheline Fromont-Racine, Andrew E. Mayes, Adeline Brunet-Simon, Jean-Christophe Rain, Alan Colley, Ian Dix, Laurence Decourty, Nicolas Joly, Florence Ricard, Jean D. Beggs, Pierre Legrain

**Affiliations:** 1 Génétique des Interactions Macromoléculaires CNRS (URA 1300) Institut Pasteur 25-28 rue du Dr Roux Paris Cedex 15 75724 France; 2 Institute of Cell and Molecular Biology University of Edinburgh, King's Buildings Mayfield Road Edinburgh EH9 3JR UK; 3 Service d'Informatique Scientifique Institut Pasteur 25-28 rue du Dr Roux Paris Cedex 15 75724 France

## Abstract

A set of seven structurally related Sm proteins forms the core of the snRNP particles containing the spliceosomal U1, U2, U4 and U5 snRNAs. A search of the genomic sequence of *Saccharomyces cerevisiae* has identified a number of open reading frames that potentially encode structurally similar proteins termed Lsm (Like Sm) proteins. With the aim of analysing all possible interactions between the Lsm proteins and any protein encoded in the yeast genome, we performed exhaustive and iterative genomic two-hybrid screens, starting with the Lsm proteins as baits. Indeed, extensive interactions amongst eight Lsm proteins were found that suggest the existence of a Lsm complex or complexes. These Lsm interactions apparently involve the conserved Sm domain that also mediates interactions between the Sm proteins. The screens also reveal functionally significant interactions with splicing factors, in particular with Prp4 and Prp24, compatible with genetic studies and with the reported association of Lsm proteins with spliceosomal U6 and U4/U6 particles. In addition, interactions with proteins involved in mRNA turnover, such as Mrt1, Dcp1, Dcp2 and Xrn1, point to roles for Lsm complexes in distinct RNA metabolic processes, that are confirmed in independent functional studies. These results provide compelling evidence that two-hybrid screens yield functionally meaningful information about protein–protein interactions and can suggest functions for uncharacterized proteins, especially when they are performed on a genome-wide scale.


**Yeast**
*Yeast* 2000; **17**: 95–110.

**Research Article**

### Genome-wide protein interaction screens reveal functional networks involving Sm-like proteins

Micheline Fromont-Racine[1†], Andrew E. Mayes[2†], Adeline Brunet-Simon[1], Jean-Christophe Rain[1], Alan Colley[2], Ian Dix[2], Laurence Decourty[1], Nicolas Joly[3], Florence Ricard[1], Jean D. Beggs[2] and Pierre Legrain[1]*

[1] *Génétique des Interactions Macromoléculaires, CNRS (URA 1300), Institut Pasteur, 25–28 rue du Dr Roux, 75724 Paris Cedex 15, France*
[2] *Institute of Cell and Molecular Biology, University of Edinburgh, King's Buildings, Mayfield Road, Edinburgh EH9 3JR, UK*
[3] *Service d'Informatique Scientifique, Institut Pasteur, 25–28 rue du Dr Roux, 75724 Paris Cedex 15, France*

**Correspondence to: P. Legrain, Génétique des Interactions Macromoléculaires, CNRS (URA 1300), Institut Pasteur, 25–28 rue du Dr Roux, 75724 Paris Cedex 15, France. E-mail: plegrain@pasteur.fr*

*†M. F.-R. and A. E. M. contributed equally to this work.*

Received: 13 March 2000
Accepted: 17 April 2000

### Abstract

**A set of seven structurally related Sm proteins forms the core of the snRNP particles containing the spliceosomal U1, U2, U4 and U5 snRNAs. A search of the genomic sequence of *Saccharomyces cerevisiae* has identified a number of open reading frames that potentially encode structurally similar proteins termed Lsm (Like Sm) proteins. With the aim of analysing all possible interactions between the Lsm proteins and any protein encoded in the yeast genome, we performed exhaustive and iterative genomic two-hybrid screens, starting with the Lsm proteins as baits. Indeed, extensive interactions amongst eight Lsm proteins were found that suggest the existence of a Lsm complex or complexes. These Lsm interactions apparently involve the conserved Sm domain that also mediates interactions between the Sm proteins. The screens also reveal functionally significant interactions with splicing factors, in particular with Prp4 and Prp24, compatible with genetic studies and with the reported association of Lsm proteins with spliceosomal U6 and U4/U6 particles. In addition, interactions with proteins involved in mRNA turnover, such as Mrt1, Dcp1, Dcp2 and Xrn1, point to roles for Lsm complexes in distinct RNA metabolic processes, that are confirmed in independent functional studies. These results provide compelling evidence that two-hybrid screens yield functionally meaningful information about protein–protein interactions and can suggest functions for uncharacterized proteins, especially when they are performed on a genome-wide scale.** **Copyright © 2000 John Wiley & Sons, Ltd.**

**Keywords:** two-hybrid; splicing; mRNA degradation; yeast; proteome; snRNP

### Introduction

Splicing of nuclear pre-mRNA occurs within a large ribonucleoprotein complex called the spliceosome. Spliceosome assembly involves snRNP particles constituted of snRNAs (the U1, U2, U4, U5 and U6 snRNAs) which are associated with proteins. Human U1, U2, U4 and U5 snRNPs contain two classes of proteins: seven small proteins, collectively called the Sm proteins (B or B′, D1, D2, D3, E, F, G) that constitute a core particle common to these snRNP, and other proteins associated specifically with one particular snRNP (Burge *et al.*, 1998; Will and Lührmann, 1997). These snRNP particles are evolutionary highly conserved and Sm proteins were also identified in the yeast *Saccharomyces cerevisiae* (Bordonné and Tarassov, 1996; Gottschalk *et al.*, 1998; Roy *et al.*, 1995; Rymond *et al.*, 1993). All Sm proteins contain two conserved regions, called the Sm motifs 1 and 2 (Cooper *et al.*, 1995; Hermann *et al.*, 1995; Seraphin 1995). The U1, U2, U4 and U5 snRNAs are transcribed by RNA polymerase II and exported to the cytoplasm, where they associate with a complex of Sm proteins

Copyright © 2000 John Wiley & Sons, Ltd.

to form core snRNP particles. These U snRNAs contain a conserved structural motif, a single-stranded uridylic acid-rich region flanked by two stem-loop structures (Branlant *et al.*, 1982), which is recognized by the Sm protein complex. When the Sm core particle is assembled, the 5′ cap of the snRNA becomes hypermethylated and 3′-end processing occurs (Jacobson *et al.*, 1993). At least in higher eukaryotes, binding of the Sm core proteins is essential for the hypermethylation of the cap (Mattaj, 1986) and both the 5′ trimethylguanosine cap and the Sm proteins are required for the nuclear import of the snRNP (Fischer *et al.*, 1993). Finally, with the addition of snRNP-specific proteins, a functional snRNP is produced. Thus, in higher eukaryotes the biogenesis of snRNPs is a complex process involving both nuclear and cytoplasmic compartments.

The U6 snRNA is different; it is transcribed by RNA polymerase III and has a $\gamma$-monomethyl triphosphate cap. U6 snRNA lacks the Sm binding site and does not itself assemble with the canonical Sm proteins (Luhrmann *et al.*, 1990). However, the U4 and U6 snRNAs have extensive sequence complementarity to one another and most or all of the U4 snRNA is found complexed with U6 snRNA in a U4/U6 di-snRNP, while a free form of U6 also exists. There have been conflicting reports about the localization of the U6 snRNA. For example, it was reported that in *Xenopus* oocytes U6 snRNA does not leave the nucleus (Vankan *et al.*, 1990), whereas work on mouse fibroblast cells indicated that newly synthesised U6 snRNA is present transiently in the cytoplasm (Fury and Zieve, 1996).

Searches in the *Saccharomyces cerevisiae* genome database allowed the identification of another set of Sm-like proteins (Fromont-Racine *et al.*, 1997). One of them, Lsm8, was identified in a two-hybrid screen using the splicing factor Hsh49p as bait (Fromont-Racine *et al.*, 1997), indicating a possible link with the splicing machinery. Indeed, seven out of these nine Sm-like proteins, renamed Lsm 2–8, were found to associate with U6 snRNA (Cooper *et al.*, 1995; Mayes *et al.*, 1999; Pannone *et al.*, 1998; Seraphin, 1995), suggesting the possible existence in budding yeast of a heptameric U6-associated Lsm particle that may be similar to the Sm core particle. Indeed, most of these Lsm proteins were found associated with the U4/U6.U5 tri-snRNP (Gottschalk *et al.*, 1999; Stevens and Abelson, 1999). In contrast, Lsm1 displays only a weak, highly salt-sensitive association with U6 (Mayes *et al.*, 1999) and yeast genetic studies have implicated Lsm1 (previously Spb8) in mRNA decapping (Boeck *et al.*, 1998). Thus, Lsm 1 protein could be involved in a distinct pathway. The last Lsm protein, SmX1 or Lsm9, does not bind to U6 snRNA and was recently found in a protein complex unrelated to splicing (Rigaut *et al.*, 1999). Little is known on the biogenesis of the U6 snRNP particle, apart from a proposed chaperone function for La protein, handing the newly synthesized U6 snRNA from RNA polymerase III to Lsm proteins (Pannone *et al.*, 1998). Also, in humans, seven Lsm orthologous proteins were found associated with U6 snRNA (Achsel *et al.*, 1999).

In order to understand what could be the various role of Lsm proteins, we used exhaustive and iterative two-hybrid screens, starting with Lsm proteins as baits. Interactions amongst the Lsm proteins themselves strongly suggest the existence of a Lsm complex or complexes. These interactions require both Sm motifs. The screens also reveal several interactions with splicing factors that may be functionally significant. In particular, the interactions with Prp4 and Prp24 are compatible with the observed association of Lsm proteins with U6 and U4/U6 particles, and with genetic studies. Interactions with SmD2, Prp11 and Hsh49 suggest that Lsm proteins may also play a key role in assembling spliceosomes through snRNP–snRNP interactions. In addition, the screens reveal interactions with proteins which are involved in mRNA turnover, hinting that Lsm1 may not be the only Lsm protein associated with such a function. Altogether, the Lsm screens and the iterative screens point to roles for Lsm proteins in distinct processes.

### Materials and methods

### Strains and plasmids

Y187, CG1945 and L40 strains were used to perform the two-hybrid screens (Fromont-Racine *et al.*, 1997). We derived the L40ΔG from the L40 strain by deleting the GAL4 gene in this strain and replacing it by a $KANA^R$ cassette (see below). Gap

Copyright © 2000 John Wiley & Sons, Ltd. 

repair experiments were performed in the yeast strain BMA64 (F. Lacroute). The *Escherichia coli* strain MC1066 was used for prey plasmid recovery, selecting on plates lacking leucine.

The pBTM116 plasmid (Vojtek *et al.*, 1993) was used to clone LexA fusions and pAS2ΔΔ was used to clone the Gal4 bait fusions (Fromont-Racine *et al.*, 1997).

### Plasmid bait constructions

The full-length ORFs were always used. For LSM1 (YJL124C) a *Bam*H1 fragment taken from a prey plasmid fused at nucleotide -52 relative to the initiation codon was cloned in-frame into a modified pBTM116 plasmid. For LSM2 (YBL026W without the intron) a *Bam*HI–*Pst*I fragment produced by reverse transcription followed by PCR amplification (Sambrook *et al.*, 1989) was cloned into pAS2ΔΔ and pBTM116 plasmids. For LSM3 (YLR438CA) a *Nco*1–*Bam*H1 PCR fragment was cloned into pAS2ΔΔ. For LSM4 (YER112W), a *Nco*1–*Bam*H1 PCR fragment was cloned into pAS2ΔΔ. For LSM5 (YER146W), a *Bam*H1 fragment taken from a prey plasmid fused at nucleotide -2 respective to the start codon was cloned in-frame into pBTM116 plasmid. For LSM6 (YDR378C) a *Eco*R1–*Bam*H1 PCR fragment was cloned in pBTM116 plasmid. For LSM7 (YNL147W without the intron) a PCR fragment was cloned into pBTM116 plasmid at the *Sma*1 site. For LSM8 (YJR022W) a *Nco*I–*Xho*I fragment from a prey plasmid fused at nucleotide -2 with respect to the initiation codon was cloned into pAS2ΔΔ between the *Nco*I and *Sal*1 sites, then subcloned as a *Bam*H1 fragment into pBTM116. For YEL015W a *Eco*R1–*Sal*1 PCR fragment was cloned into pAS2ΔΔ. All sequences derived by PCR cloning were verified. PSU1 was cloned in pAS2ΔΔ by gap repair, using two PCR fragments with about 200 nucleotides of homology to the 5′ and 3′ ends of the gene cloned in pAS2ΔΔ. After gap repair, the plasmid was recovered and the gene was checked by restriction mapping and sequencing of the 5′ end. PAT1 full-length sequence as bait had very high autoactivating activity, so a pGBT9 derivative bait plasmid lacking the highly acidic N-terminal 51 amino acids was used for the screen (a generous gift from F. Lacroute).

### L40ΔG strain construction and two-hybrid mating

Two-hybrid screens were performed by a mating strategy, using the FRYL library introduced in Y187 cells and either CG1945 cells producing Gal4-derived bait proteins or L40 and L40ΔG cells producing LexA-derived bait proteins (Fromont-Racine *et al.*, 1997). For the LexA–Lsm6 and LexA–Lsm8 screens, only the HIS3 reporter was used to select interactors (diploid cells derived with L40 cells express endogenous Gal4 gene, which spontaneously activates the LacZ reporter gene from the Y187 background). To permit use of the LacZ reporter with other LexA baits, we generated the strain L40ΔG by replacing the entire *GAL4* coding sequence in L40 by the kanamycin-resistance marker from plasmid pkana-X2 (Wach *et al.*, 1994). The gene replacement was confirmed by Southern blotting, and an X-gal overlay on diploid cells (Y187 × L40ΔG) verified that endogenous activation of the LacZ reporter did not occur (data not shown).

### Selection of positive clones

Positive clones were selected as previously described (Fromont-Racine *et al.*, 1997). Prey inserts were amplified from library plasmids by PCR on colonies (Wang *et al.*, 1996a), the length of each insert was determined by gel electrophoresis and the 5′ junction was sequenced. Identification of each candidate in the yeast database was performed by a dedicated software (DOGEL) that gives the chromosomal coordinates (chromosome number, strand and position), ORF and gene name and the exact location of the beginning of the insert relative to the initiation codon. Alternatively, the BLAST program can be used against the Saccharomyces Genome Database (SGD; http://genome-www.stanford.edu). The biological information on the ORF was extracted from the Yeast Protein Database (YPD; http://www.proteome.com).

### Classification of the candidates

Prey fusions were classified according to four different categories of different heuristic values (A1 > A2 = A3 > A4) (Fromont-Racine *et al.*, 1997). B fusions express non-biological peptides, i.e anti-sense or intergenic regions, and are excluded from the tables of results.

Copyright © 2000 John Wiley & Sons, Ltd. 

### Results

### Characteristics of genomic interaction screens with Lsm proteins

With the aim of identifying as many as possible of the proteins that interact with Lsm proteins we performed exhaustive and iterative two-hybrid screens using the FRYL *S. cerevisiae* genomic library (Fromont-Racine *et al.*, 1997) (Table 1; see Materials and methods). For each screen, results are analysed in order to evaluate the heuristic value of each prey protein (Fromont-Racine *et al.*, 1997) (see also Materials and methods). In addition, the domain of interaction selected for each prey protein is identified (Table 1). Figure 1 presents the characteristics of the screens, showing for each bait protein the number of ORFs selected in each category, and the number of clones of each ORF. Comparing the profiles for different bait proteins, it is apparent that, despite the similarity of structure between Lsm proteins, they behave differently in the screens. Some, such as Lsm7, have few partners, whereas others, such as Lsm1, have many partners.

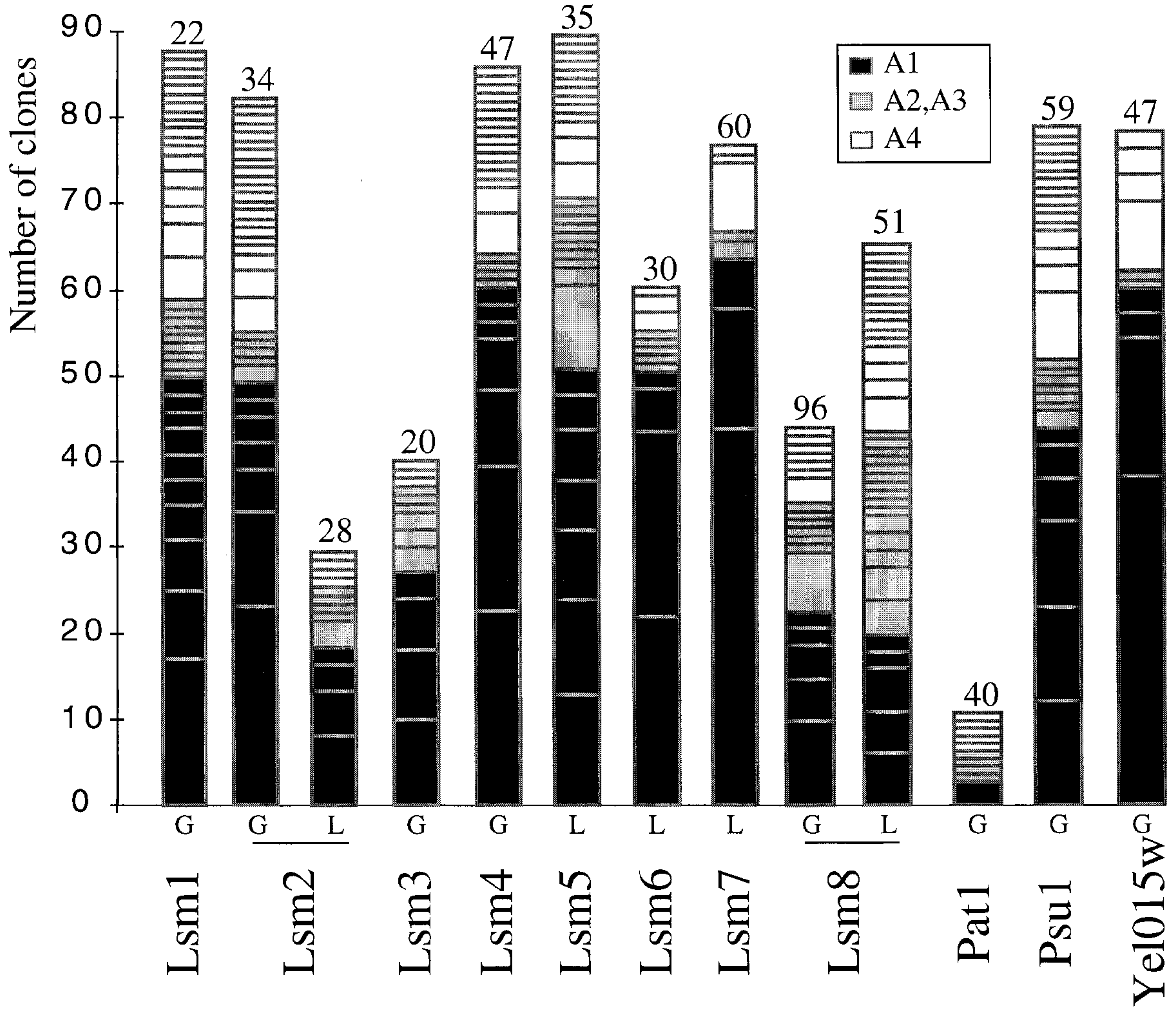

**Figure 1.** Distribution profiles of prey proteins found in two-hybrid screens. Each histogram represents one given screen with a Gal4 (G) or a LexA (L) fusion-bait cloned into pAS2$^{-}$ and pBTM116 plasmids, respectively. Each box corresponds to one ORF prey and is drawn according to categories (Fromont-Racine *et al.*, 1997) (see insert and Materials and methods). The size of the box represents the number of clones related to one particular ORF. The number of interactions tested in each screen is indicated on the top of the histogram (in millions)

Copyright © 2000 John Wiley & Sons, Ltd. 

Table 1. Results of genomic protein interaction screens

| Bait | Prey ORF name (a) | Prey gene name (b) | Start domain (c) | End domain (d) | Cat (e) | Clones number (f) |
|---|---|---|---|---|---|---|
| Lsm1-Gal4 | YAL019W | FUN30 | 432 | 950 | A3 | 1 |
| | YBL026W | LSM2 | 2 | end | A1 | 8(3) |
| | YBR214W | SDS24 | 282 | end | A4 | 1 |
| | YBR274W | CHK1 | 355 | end | A4 | 4 |
| | YCR024C | | 271 | end | A4 | 1 |
| | YCR077C | PAT1 | 95;353(g) | 350;end(g) | A1 | 17(5) |
| | YDL013W | HEX3 | 6 | 350 | A2 | 1 |
| | YDL175C | | 1 | end | A2/A3 | 1 |
| | YDR002W | YRB1 | 39 | end | A2 | 1 |
| | YDR378C | LSM6 | 54 | end | A1 | 2(2) |
| | YDR422C | SIP1 | 473 | end | A4* | 1 |
| | YEL060C | PRB1 | 58 | 300 | A1 | 3(3) |
| | YER028C | | 308 | end | A4* | 2 |
| | YGL207W | SPT16 | 813 | end | A1* | 4(2) |
| | YGR158C | MTR3 | 44 | end | A2 | 1 |
| | YIL042C | | 14 | end | A2/A3 | 1 |
| | YIL048W | NEO1 | 1048 | end | A4* | 5 |
| | YJR143C | PMT4 | 661 | end | A4* | 2 |
| | YKL173W | SNU114 | 441 | 600 | A4 | 1 |
| | YKR026C | GCN3 | 62 | 200 | A1 | 3(2) |
| | YLR003C | | 214 | end | A1 | 6(3) |
| | YLR362W | STE11 | 172 | 350 | A4 | 2 |
| | YLR438C-A | LSM3 | 1 | end | A2* | 1 |
| | YML088W | | 165 | end | A1 | 2(2) |
| | YMR056C | AAC1 | 108 | end | A4* | 1 |
| | YMR250W | | 418 | end | A4 | 1 |
| | YNL032W | SIW14 | 46 | end | A4 | 1 |
| | YNL163C | EF4 | 97 | 250 | A4 | 1 |
| | YNL276C | | 128 | end | A4 | 1 |
| | YOR109W | INP53 | 167 | 300 | A4 | 1 |
| | YOR147W | | 88 | 400 | A4 | 2 |
| | YOR320C | | 424 | end | A4 | 1 |
| | YOR375C | GDH1 | 42 | end | A2/A3 | 1 |
| | YPL016W | SWI1 | 558 | 900 | A1 | 2(2) |
| | YPL084W | BRO1 | 1 | 200 | A2 | 1 |
| | YPL152W | RRD2 | 329 | end | A4* | 1 |
| | | Ty2-1 | ND | ND | A1 | 3(2) |
| Lsm2-Gal4 | YAR003W | FUN16 | 46 | 150 | A4* | 1 |
| | YBL066C | SEF1 | 446 | 700 | A4 | 1 |
| | YCR020C-A | SMX1 | 1 | end | A2 | 1 |
| | YCR066W | RAD18 | 155 | end | A3 | 1 |
| | YCR077C | PAT1 | 353 | end | A1 | 3(2) |
| | YDL175C | | 81 | 150 | A1 | 5(2) |
| | YDR440W | DOT1 | 121 | 450 | A4 | 1 |
| | YEL015W | | 232 | end | A1 | 11(5) |
| | YER025W | GCD11 | 10 | 300 | A2* | 1 |
| | YGL173C | XRN1 | 890 | end | A3 | 1 |
| | YGL185C | | 273 | end | A4* | 1 |
| | YGR077C | PEX8 | 366 | end | A4 | 1 |
| | YIL048W | NEO1 | 1048 | end | A4* | 4 |
| | YIL066C | RNR3 | 383 | 600 | A4 | 3 |
| | YIL132C | | 134 | end | A4 | 1 |
| | YLR039C | RIC1 | 994 | end | A4 | 1 |
| | YLR120C | YPS1 | 10 | 200 | A1 | 3(2) |
| | YLR126C | | 165 | end | A1 | 2(2) |

Copyright © 2000 John Wiley & Sons, Ltd.

Table 1. continued

| Bait | Prey ORF name (a) | Prey gene name (b) | Start domain (c) | End domain (d) | Cat (e) | Clones number (f) |
|---|---|---|---|---|---|---|
| | YMR066W | SOV1 | 93 | 350 | A4 | 1 |
| | YMR207C | HFA1 | 733 | 950 | A4 | 1 |
| | YMR237W | | 234 | 400 | A1 | 2(2) |
| | YMR268C | PRP24 | 57 | end | A3 | 2 |
| | YNL118C | DCP2 | ND | ND | A1 | 23(8) |
| | YOL102C | TPT1 | 167 | end | A4 | 1 |
| | YOL163W | | 106 | end | A4 | 1 |
| | YOR017W | PET127 | 51 | 300 | A4 | 1 |
| | YOR043W | WHI2 | 240 | end | A4* | 2 |
| | YOR191W | RIS1 | 506 | 900 | A4* | 1 |
| | YPL042C | SSN3 | 403 | end | A4 | 1 |
| | YPL115C | BEM3 | 606 | 850 | A4 | 1 |
| | YPL119C | DBP1 | 291 | 500 | A4 | 1 |
| | YPL249C | | 293 | 550 | A4 | 1 |
| | YPR184W | | 1371 | end | A4 | 1 |
| Lsm2-LexA | YDR166C | SEC5 | 895 | end | A4 | 1 |
| | YJL124C | LSM1 | 1 | end | A2 | 3 |
| | YJL157C | FAR1 | 209 | 600 | A4 | 1 |
| | YJR022W | LSM8 | 23 | end | A1 | 8(3) |
| | YLR120C | YPS1 | 6 | 250 | A2 | 1 |
| | YLR128W | | 157 | end | A4* | 1 |
| | YLR275W | SmD2 | 12 | end | A1 | 2(2) |
| | YLR438C-A | LSM3 | 1 | end | A2* | 1 |
| | YNL264C | PDR17 | 266 | end | A1 | 5(3) |
| | YNL287W | SEC21 | 222 | 500 | A4 | 1 |
| | YOR017W | PET127 | 6 | 300 | A2 | 1 |
| | YPL249C | | 258 | 550 | A1 | 3(2) |
| | YPR032W | SRO7 | 15 | 300 | A2 | 1 |
| Lsm3-Gal4 | YBR108W | | 706 | end | A4 | 1 |
| | YCR066W | RAD18 | 126 | end | A4 | 1 |
| | YCR077C | PAT1 | 168;353(g) | 350;end(g) | A1 | 10(5) |
| | YDL013W | HEX3 | 4 | 400 | A2 | 1 |
| | YDL240W | LRG1 | 543 | 750 | A4 | 1 |
| | YJR022W | LSM8 | 22 | end | A1 | 6(4) |
| | YJR138W | | 1135 | 1500 | A1 | 3(2) |
| | YKR099W | BAS1 | 57 | 750 | A3 | 3 |
| | YLR067C | PET309 | 41 | 450 | A1 | 8(3) |
| | YLR281C | | 50 | end | A2 | 2 |
| | YMR142C | RPL13B | 1 | 100 | A2 | 1 |
| | YOR096W | RP30 | 1 | end | A2 | 1 |
| | YPL084W | BRO1 | 1 | 200 | A2 | 2 |
| Lsm4-Gal4 | YBR289W | SNF5 | 360 | 650 | A4 | 1 |
| | YCR077C | PAT1 | 353 | end | A4 | 1 |
| | YDL043C | PRP11 | 66 | end | A4 | 5 |
| | YDL145C | COP1 | 85 | 350 | A4 | 1 |
| | YDR082W | STN1 | 441 | end | A1 | 6(3) |
| | YDR289C | | 72 | 250 | A4 | 1 |
| | YDR386W | MUS81 | 112 | 500 | A4 | 1 |
| | YDR452W | | 61 | 350 | A1 | 2(2) |
| | YDR485C | | 664 | end | A1 | 2(2) |
| | YER124C | | 468 | end | A4 | 1 |
| | YGL173C | XRN1 | 863 | 1500 | A3 | 1 |
| | YIL029C | | 132 | end | A4 | 3 |
| | YJL110C | GZF3 | 397 | end | A1 | 2(2) |

Copyright © 2000 John Wiley & Sons, Ltd.

Table 1. continued

| Bait | Prey ORF name (a) | Prey gene name (b) | Start domain (c) | End domain (d) | Cat (e) | Clones number (f) |
|---|---|---|---|---|---|---|
| | YJL155C | FBP26 | 145 | end | A1 | 9(4) |
| | YJR022W | LSM8 | 1 | ND | A2 | 1 |
| | YJR138W | | 1004 | 1150 | A4 | 1 |
| | YKL209C | STE6 | 1012 | 1250 | A4 | 1 |
| | YLL032C | | 566 | 800 | A4 | 1 |
| | YLR275W | SmD2 | 54 | end | A4 | 1 |
| | YLR386W | | 378 | 800 | A1 | 23(9) |
| | YNL091W | | 1160 | end | A4 | 1 |
| | YNL118C | DCP2 | ND | ND | A1 | 17(4) |
| | YNL199C | GCR2 | 272 | 500 | A4 | 1 |
| | YOL004W | SIN3 | 492 | 650 | A3 | 1 |
| | YOL149W | DCP1 | 1 | end | A2 | 1 |
| | YOR195W | SLK19 | 268 | 500 | A4 | 1 |
| | YOR219C | STE13 | 366 | 500 | A4 | 1 |
| Lsm5-LexA | YBL026W | LSM2 | 1 | end | A1 | 6(2) |
| | YCR024C | | 271 | end | A4 | 1 |
| | YCR077C | PAT1 | 132;353(g) | 350;end(g) | A1 | 13(5) |
| | YDL112W | TRM3 | 889 | 1100 | A1* | 4(2) |
| | YDR110W | FOB1 | 456 | end | A4 | 1 |
| | YDR378C | LSM6 | 1 | end | A2* | 10 |
| | YER025W | GCD11 | 10 | 350 | A2* | 1 |
| | YER112W | LSM4 | 1 | end | A2 | 2 |
| | YER131W | RPS26b | 37 | end | A2* | 1 |
| | YFL066C | | 1 | end | A4 | 1 |
| | YGR210C | | 321 | end | A1 | 6(3) |
| | YHL008C | | 260 | 550 | A1* | 8(2) |
| | YIL038C | NOT3 | 609 | end | A4 | 2 |
| | YIL048W | NEO1 | 1048 | end | A4* | 4 |
| | YJL084C | | 494 | 850 | A4 | 1 |
| | YJL124C | LSM1 | 1 | end | A2 | 1 |
| | YJR138W | | 57 | 250 | A4 | 1 |
| | YKL209C | STE6 | 1012 | 1250 | A4 | 1 |
| | YKR026C | GCN3 | 39 | end | A2 | 1 |
| | YLR058C | SHM2 | 383 | end | A4 | 1 |
| | YLR275W | SmD2 | 9 | end | A2 | 1 |
| | YLR438C-A | LSM3 | 1 | end | A2* | 1 |
| | YMR268C | PRP24 | 280 | end | A1 | 3(2) |
| | YNL147W | LSM7 | 46 | end | A4 | 1 |
| | YOR201C | PET56 | 26 | 300 | A2 | 1 |
| | YOR320C | | 440 | end | A1 | 11(5) |
| | YPL090C | RPS6A | 336 | end | A4* | 1 |
| | YPL152W | RRD2 | 329 | end | A4* | 3 |
| | YPR010C | RPA135 | 656 | 1150 | A3 | 1 |
| | YPR184W | | 697 | 1035 | A4 | 1 |
| Lsm6-LexA | YBL026W | LSM2 | 1 | end | A2 | 1 |
| | YCR077C | PAT1 | 353 | end | A1 | 22(2) |
| | YER112W | LSM4 | 1 | end | A1 | 2(2) |
| | YGL251C | HFM1 | 759 | end | A4 | 2 |
| | YJL139C | YUR1 | 197 | ND | A4 | 1 |
| | YJR022W | LSM8 | 23 | end | A1 | 22(7) |
| | YLR053C | | 45 | end | A2 | 1 |
| | YLR275W | SmD2 | 47 | end | A1 | 5(3) |
| | YLR438C-A | LSM3 | 1 | end | A2 | 1 |
| | YMR221C | | 380 | end | A3 | 1 |
| | YMR268C | PRP24 | 114 | end | A4 | 2 |

Copyright © 2000 John Wiley & Sons, Ltd. 

Table 1. continued

| Bait | Prey ORF name (a) | Prey gene name (b) | Start domain (c) | End domain (d) | Cat (e) | Clones number (f) |
|---|---|---|---|---|---|---|
| | YNL147W | LSM7 | 1 | end | A2 | 1 |
| | YOR320C | | 404 | end | A4 | 2 |
| Lsm7-LexA | YCR077C | PAT1 | 153 | 350 | A1 | 14(7) |
| | YDL077C | VAM6 | 402 | 600 | A4* | 8 |
| | YFL066C | | 1 | end | A4 | 1 |
| | YIL112W | | 1043 | end | A1 | 44(14) |
| | YLR438C-A | LSM3 | 1 | end | A2 | 2 |
| | YMR268C | PRP24 | 354 | end | A4 | 1 |
| | YPR178W | PRP4 | 109 | end | A3 | 1 |
| | | Ty2-1 | ND | ND | A1 | 6(4) |
| Lsm8-Gal4 | YBL026W | LSM2 | 2 | end | A1 | 4(3) |
| | YBR003W | COQ1 | 251 | end | A4 | 1 |
| | YCR077C | PAT1 | 426 | end | A4* | 1 |
| | YDR228C | PCF11 | 272 | 550 | A1 | 2(2) |
| | YDR277C | MTH1 | 22 | end | A2/A3 | 1 |
| | YEL015W | | 232 | end | A1 | 5(3) |
| | YER112W | LSM4 | 12 | end | A2 | 1 |
| | YGL096W | | 73 | end | A1 | 2(2) |
| | YGL173C | XRN1 | 1123 | 1500 | A4 | 1 |
| | YGR158C | MTR3 | 3 | end | A2 | 1 |
| | YHR034C | | 272 | end | A4 | 1 |
| | YHR035W | | 18 | 200 | A2 | 1 |
| | YIL173W | VTH1 | 437 | 650 | A4 | 1 |
| | YNL050C | | 1 | 200 | A4* | 3 |
| | YNL118C | DCP2 | ND | ND | A1 | 10(5) |
| | YNR050C | LYS9 | 264 | end | A4 | 1 |
| | YNR053C | | 1 | end | A2/A3 | 7 |
| | YOR076C | | 196 | end | A3 | 1 |
| | YOR319W | HSH49 | 16 | end | A2 | 1 |
| Lsm8-LexA | YBL026W | LSM2 | 2 | end | A2* | 1 |
| | YBR034C | HMT1 | 15 | 200 | A2 | 1 |
| | YBR044C | | 492 | end | A1 | 2(2) |
| | YCR020C-A | SMX1 | 1 | end | A2 | 2 |
| | YCR077C | PAT1 | 353 | end | A1 | 6(2) |
| | YCR107W | AAD3 | 49 | end | A4* | 1 |
| | YDR127W | ARO1 | 1 | 150 | A2 | 1 |
| | YDR135C | YCF1 | 1445 | end | A4 | 1 |
| | YDR184C | ATC1 | 62 | end | A4 | 1 |
| | YDR228C | PCF11 | 289 | 500 | A4 | 2 |
| | YDR378C | LSM6 | 1 | end | A2* | 4 |
| | YEL023C | | 1 | 200 | A2* | 1 |
| | YER146W | LSM5 | 9 | end | A1 | 2(2) |
| | YGL028C | SCW11 | 129 | 300 | A4* | 1 |
| | YGL096W | | 73 | end | A4 | 1 |
| | YGR158C | MTR3 | 3 | end | A2 | 1 |
| | YHR035W | | 18 | 200 | A2 | 1 |
| | YJL004C | SYS1 | 136 | end | A4* | 2 |
| | YJL021C | | 129 | end | A4 | 1 |
| | YKR021W | | 1 | 200 | A2 | 2 |
| | YKR104W | | 219 | end | A4 | 1 |
| | YLL015W | | 1495 | end | A4 | 1 |
| | YLR133W | CKI1 | 63 | 250 | A4 | 1 |
| | YLR143W | | 614 | end | A1 | 5(4) |
| | YLR430W | SEN1 | 971 | 1200 | A4* | 4 |

Copyright © 2000 John Wiley & Sons, Ltd.

Table 1. continued

| Bait | Prey ORF name (a) | Prey gene name (b) | Start domain (c) | End domain (d) | Cat (e) | Clones number (f) |
|---|---|---|---|---|---|---|
| | YLR438C-A | LSM3 | 1 | end | A2* | 4 |
| | YMR205C | PFK2 | 2 | 350 | A2 | 1 |
| | YMR268C | PRP24 | 114 | end | A3 | 1 |
| | YNL227C | | 214 | 450 | A4 | 1 |
| | YNL242W | | 1273 | end | A4 | 2 |
| | YNL329C | PEX6 | 677 | end | A3 | 2 |
| | YOL031C | | 55 | 400 | A4* | 1 |
| | YOL140W | ARG8 | 165 | 400 | A4* | 1 |
| | YOR076C | | 196 | end | A3 | 1 |
| | YOR319W | HSH49 | 16 | end | A1 | 5(2) |
| | YPR178W | PRP4 | 53 | end | A3 | 1 |
| Pat1(Ycr077c) | YDR141C | | 454 | 800 | A4 | 1 |
| | YDR389W | SAC7 | 466 | end | A4 | 1 |
| | YER115C | SPR6 | 157 | end | A4 | 1 |
| | YGL143C | MRF1 | 336 | end | A4 | 1 |
| | YML109W | ZDS2 | 615 | end | A4 | 1 |
| | YMR002W | | 1 | end | A4 | 1 |
| | YMR288W | | 472 | 700 | A4 | 1 |
| | YNL118C | PSU1 | ND | ND | A1 | 3(3) |
| | YNR027W | | 34 | end | A2 | 1 |
| | YNR053C | | ND | ND | A4 | 1 |
| Ye1015w | YBL061C | SKT5 | 254 | 500 | A4 | 3 |
| | YBL066C | SEF1 | 446 | 700 | A4* | 2 |
| | YEL015W | | 238 | end | A1 | 16(5) |
| | YER032W | FIR1 | 710 | end | A1 | 3(2) |
| | YER124C | | 468 | end | A4 | 3 |
| | YGL173C | XRN1 | 864 | end | A3 | 1 |
| | YGR116W | SPT6 | 1103 | end | A3 | 1 |
| | YJR140C | HIR3 | 1574 | end | A1* | 3(2) |
| | YLR082C | SRL2 | 91 | 350 | A4 | 8 |
| | YNL118C | PSU1 | ND | ND | A1 | 38(10) |
| Psu1(Ynl118c) | YAR009C | | 966 | 1150 | A1 | 2(2) |
| | YBL034C | STU1 | 1 | 350 | A2 | 1 |
| | YBL037W | APL3 | 517 | 800 | A4 | 1 |
| | YBL045C | COR1 | 335 | end | A4 | 1 |
| | YBL054W | | 161 | 450 | A4* | 2 |
| | YCR076C | | 232 | end | A4* | 1 |
| | YDL116W | NUP84 | 12 | 300 | A2 | 1 |
| | YDR472W | | 102 | end | A4 | 1 |
| | YEL015W | | 232 | end | A1 | 11(5) |
| | YGL014W | | 653 | end | A4* | 1 |
| | YGL049C | TIF4632 | 663 | 900 | A4 | 1 |
| | YGL173C | XRN1 | 890 | 1450 | A1 | 4(2) |
| | YGR116W | SPT6 | 1103 | end | A3 | 1 |
| | YHR186C | | 680 | 1350 | A3 | 1 |
| | YIR014W | | 81 | 250 | A4 | 1 |
| | YIR024C | GIF1 | 31 | end | A1 | 10(3) |
| | YJR023C | | 3 | end | A2 | 1 |
| | YKL133C | | 230 | end | A4 | 2 |
| | YKR031C | SPO14 | 1372 | end | A1 | 12(2) |
| | YKR054C | DYN1 | 201 | 550 | A4 | 3 |
| | YLL001W | DNM1 | 535 | end | A4 | 1 |
| | YLR451W | LEU3 | 413 | 750 | A4 | 1 |
| | YML099C | ARG81 | 659 | end | A4 | 1 |

Copyright © 2000 John Wiley & Sons, Ltd. 

Table 1. continued

| Bait | Prey ORF name (a) | Prey gene name (b) | Start domain (c) | End domain (d) | Cat (e) | Clones number (f) |
|---|---|---|---|---|---|---|
| | YML112W | CTK3 | 1 | 200 | A2 | 2 |
| | YOL151W | GRE2 | 101 | end | A1 | 5(2) |
| | YOR023C | | 31 | 250 | A4 | 8 |
| | YOR093C | | 932 | 1400 | A3 | 1 |
| | YOR124C | UBP2 | 567 | 850 | A4 | 1 |
| | YPR160W | GPH1 | 545 | 750 | A4 | 1 |

(a) All the ORFs found in each screen are listed.
(b) A gene name is given when available.
(c) The N-Terminal residue of the smallest overlapping fragment is precisely located by sequencing.
(d) The C-Terminal extremity of the smallest overlapping fragment is roughly located according to PCR fragment sizes.
(e) The out-of-frame fusions are noted by an asterisk. For A1 candidates, an asterisk indicates those for which all fusions were in the same alternative frame.
(f) The total number of clones is indicated as well as the number of different fusions (in brackets) for A1 candidates.
(g) In those cases, two different non-overlapping domains are identified.
A2/A3: stands for candidates having both A2 and A3 characteristics (Fromont-Racine *et al.*, 1997).
ND, not determined.

Two Lsm proteins (Lsm2 and Lsm8) were used as both Gal4 and LexA fusion proteins (Table 1). Some of the prey proteins were found in common and represent a subset of highly specific interactors. In addition, for both Lsm2 and Lsm8 proteins, highly significant prey proteins (A1 category) were found with either the Gal4 or the LexA bait but not with both. As Lsm proteins are relatively small, the construction of fusions may be more likely to cause folding problems or steric interference with protein–protein interactions, as interacting domains may be masked in the fusion protein. In theory, saturating two-hybrid screens should identify multiple samples of the same partner. According to this criterion, the larger the screen the greater is the probability that single clones will be non-specific because their occurrence most probably reflects sporadic selection. For example, in the Lsm7 screen (60 million interactions tested, only eight distinct genetic loci as prey ORFs, out of them three found as single clones) or in the Lsm8–Gal4 screen (96 million interactions, 19 distinct genetic loci, 12 single clones) single clones might be considered likely to be non-specific, whereas in the rather small-scale Lsm1 screen (22 million interactions, 37 different genetic loci, 21 single clones) single clones may be more significant. When the data from several functionally related screens are pooled, prey found as single clones in several screens become more significant. For example, Prp24 arose as single isolates with Lsm7 and Lsm8 and became more significant when the data were combined, especially because this protein had never been selected in more than 100 previous screens (data not shown; see Discussion). Xrn1 was found only as single clones in three screens, which might suggest indirect or transient interactions that would be statistically less likely to be observed, but are still meaningful. The 8 Lsm baits produced 229 interactions with 161 different prey (Table 1). Among all these interactions, 25 are connections between Lsm proteins. Thus it is noticeable that more than 15% of the ORFs found in the screens performed with Lsm proteins correspond to these eight small Lsm genes (less than 600 nucleotides) selected out of a collection of more than 6000 genes covering more than 15 megabases. These results reveal very likely interactions between these proteins.

### Connections of Lsm proteins with each other

Connections between the Lsm proteins are shown in Figure 2A. All eight Lsm proteins interacted with at least three other Lsm proteins, although not all interactions were found reciprocally. All these 25 pairings involved highly significant interactions (A1 or A2 categories; Fromont-Racine *et al.*, 1997). It is particularly striking that all selected prey fragments start near the natural N-terminus and all contain both Sm motifs. These results strongly suggest that the interactions between the Lsm proteins require the Sm motifs, and deletion analysis demonstrated

Copyright © 2000 John Wiley & Sons, Ltd.

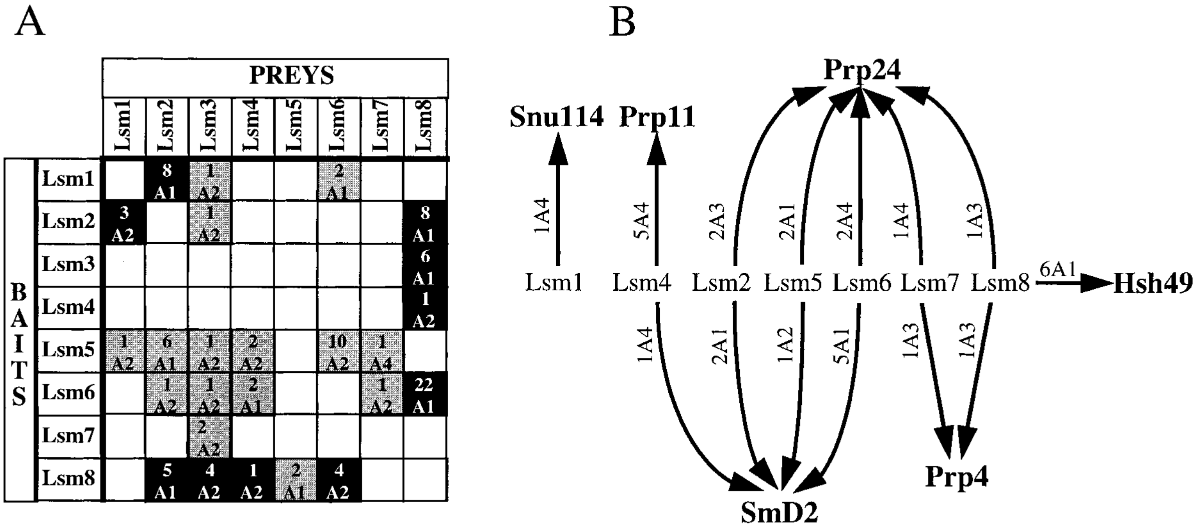

**Figure 2.** Lsm proteins and splicing. (A) Lsm proteins interact together. Each line corresponds to one screen. Grey squares correspond to directional interactions, black squares to bidirectional interactions. For each prey the number of clones is indicated above the category. (B) Links between Lsm proteins and known mRNA splicing factors. Interactions found between Lsm proteins and known mRNA splicing factors are representated by arrows. For each interaction the number of clones is indicated this to be the case for Lsm4 (Mayes *et al.*, 1999). The multiple connections shown in Figure 2A might suggest that the Lsm proteins interact promiscuously with each other. However, no homotypic interactions were found for the Lsm proteins, indicating that these interactions did not occur spuriously between any protein-bearing Sm motifs.

### Interactions with other splicing factors

With the exception of Lsm3, all the Lsm proteins made connections with known pre-mRNA splicing factors in these screens, with a total of six splicing factors being selected as prey (Figure 2B). Lsm1 found only one, Snu114, a U5 snRNP-specific protein, while each of the others interacted with at least two. This interaction has a low predictive value, because the Snu114 prey protein has been selected only once as an A4 candidate (see Materials and methods). Lsm8, which was the most interactive amongst the Lsm proteins, is also the most connected with splicing factors. Two splicing factors specifically associated with the U2 snRNP were selected with the Lsm proteins; Prp11 was found with Lsm4, and Hsh49 with Lsm8. Significantly, in a previous screen the reciprocal interaction of Lsm8 with Hsh49 as bait was found (Fromont-Racine *et al.*, 1997). Another splicing factor that arose frequently in these screens is one of the canonical Sm proteins, SmD2, being found with Lsm2, Lsm4, Lsm5 and Lsm6. Lsm7 and Lsm8 both selected the splicing factor Prp4, which is also a component of U4–U6 snRNPs and U4–U6–U5 tri-snRNPs (Banroques and Abelson, 1989; Bjorn *et al.*, 1989). Links between Lsm proteins and SmD2 and Prp4 are in agreement with the identification of the proteins of the yeast tri-snRNP (Gottschalk *et al.*, 1999). Prp24 arose most frequently in the screens, interacting with Lsm2, Lsm5, Lsm6, Lsm7 and Lsm8 (Figure 2B). Prp24 is an RNA-binding protein that associates with U6 snRNA in free U6 snRNP and U4–U6 di-snRNP particles (Ghetti *et al.*, 1995; Jandrositz and Guthrie, 1995). The high occurrence of Prp24 in these screens could be therefore indicative of a functional interaction of the Lsm proteins with the U6 and/or U4–U6 particles, which is further supported by genetic tests. Overproduction of the Prp24 protein partially complements the growth defect of cells metabolically depleted of Lsm4, whereas overexpression of Lsm4 exacerbates the temperature sensitivity of *prp24-1* cells (AEM, M. Cooper and JDB unpublished results). It is noteworthy that Prp24, Prp4 and SmD2 have not been selected as prey in exhaustive screens that have been performed with dozens of splicing factors in our

Copyright © 2000 John Wiley & Sons, Ltd.

laboratories (Fromont-Racine *et al.*, 1997; unpublished results), thus supporting the likely functional significance of their interactions with the Lsm proteins.

### Connections with other factors

Additional prey proteins, which are neither Lsm proteins nor known splicing factors, arose multiple times in Lsm screens (Figure 3A). Three prey proteins, Pat1, Psu1 and Yel015w, show the most frequent as well as significant links with the Lsm proteins. Yel015w was found interacting with Lsm2 and Lsm8 as many independent fusions (Table 1). Similarly, Psu1 was found interacting with Lsm2, Lsm4 and Lsm8, whereas Pat1 was found as prey by each of the Lsm proteins (Figure 3A). Additional prey proteins with a high predictive value were found that might have a biological significance (see Discussion): Xrn1, a 5′>3′ exonuclease that represents the major nuclease activity for the degradation of decapped mRNAs (Jacobs *et al.*, 1998; Johnson, 1997); Gcn 3 and Gcd11, two translational initiation factors (Erickson *et al.*, 1997; Pavitt *et al.*, 1998) and Mtr3, a component of the exosome (Allmang *et al.*, 1999). All these interactions with the Lsm proteins seem very specific, since prey proteins were all A1, A2 or A3 candidates that were specifically selected by at least two Lsm bait proteins and were otherwise not found in more than 100 genomic screens done in our laboratories (Fromont-Racine *et al.*, 1997); unpublished results). The three proteins Yel015w, Pat1 and Psu1 were used in turn as bait proteins to screen the yeast proteome for potential interacting partners (Table 1, Figure 1). Curiously, none of the Lsm proteins was found in these second-round screens. Nevertheless, the complete set of connections identified through exhaustive two-hybrid screens performed with those novel proteins associate the Lsm proteins with a group of proteins that are related to the mRNA degradation pathway (Figure 3B; see Discussion).

A

| BAITS | Ycr077c Pat1/Mrt1 | Ynl118c Psu1/Dcp2 | Yel015w | Yjr138w | Yor320c | Ydl175c | Ygl173c Xrn1 | Ykr026c Gcn3 | Ycr020ca SmX1 | Ygr158c Mtr3 | Ypl084w Bro1 | Ydl013w Hex3 | Yer025w Gcd11 |
|---|---|---|---|---|---|---|---|---|---|---|---|---|---|
| Lsm1 | 17 A1 | | | | 1 A4 | 1 A2 | | 3 A1 | | 1 A2 | 1 A2 | 1 A2 | |
| Lsm2 | 3 A1 | 23 A1 | 11 A1 | | | 5 A1 | 1 A3 | | 1 A2 | | | | 1 A2 |
| Lsm3 | 10 A1 | | | 3 A1 | | | | | | | 2 A2 | 1 A2 | |
| Lsm4 | 1 A4 | 17 A1 | | 1 A4 | | | 1 A3 | | | | | | |
| Lsm5 | 13 A1 | | | 1 A4 | 11 A1 | | | 1 A2 | | | | | 1 A2 |
| Lsm6 | 22 A1 | | | | 2 A4 | | | | | | | | |
| Lsm7 | 14 A1 | | | | | | | | | | | | |
| Lsm8 | 7 A1 | 10 A1 | 5 A1 | | | | 1 A4 | | 2 A2 | 2 A2 | | | |

B

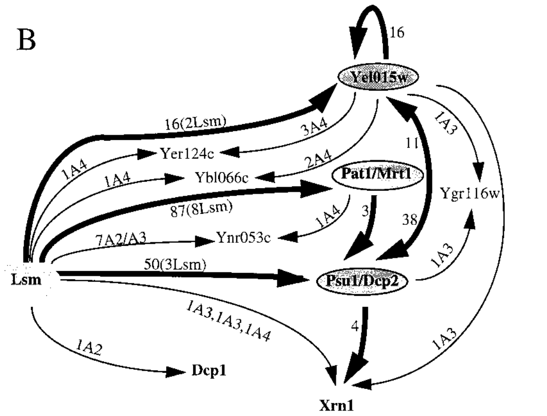

**Figure 3.** Lsm proteins and the mRNA degradation pathway. (A) Proteins connected to Lsm proteins. The most significant proteins found as prey, with at least two different Lsm proteins and which are neither Lsm proteins nor known mRNA splicing factors, are classified from right to left according to their increasing heuristic value (Fromont-Racine *et al.*, 1997). For each interaction the number of clones is indicated above the category. (B) Interaction network of proteins involved in mRNA degradation pathway. All direct and indirect (via another protein) connections between Lsm proteins and mRNA degration factors are indicated. Thick lines with arrowheads represent links of high heuristic value (A1). Other links are represented by thin lines. The category, the number of clones and the number of different Lsm bait proteins that selected a given prey are indicated

### Discussion

The multiple interactions among the eight Lsm proteins strongly suggest the existence of a complex or complexes of Lsm proteins. The interactions of Lsm1 with Lsm2, Lsm3, Lsm5 and Lsm6 seemed surprising initially, in view of the evidence that Lsm2, Lsm3, Lsm4, Lsm5, Lsm6, Lsm7 and Lsm8 associate with, and stabilize, the spliceosomal U6 snRNA (Mayes *et al.*, 1999; Salgado-Garrido *et al.*, 1999), whereas Lsm1 appears to be involved in a distinct process, mRNA decapping (Boeck *et al.*, 1998). Also, a seven- (rather than eight)-component complex of U6-associated Lsm proteins is attractive in view of the heptameric complex predicted for the human Sm proteins. Indeed, Lsm2–Lsm8 proteins have recently been identified in yeast and human

Copyright © 2000 John Wiley & Sons, Ltd. 

cells spliceosomes (Achsel *et al.*, 1999; Gottschalk *et al.*, 1999; Stevens and Abelson, 1999). It is not yet established how similar the canonical Sm and the Lsm2–Lsm8 complexes are. It should be noticed that we found Lsm proteins highly connected to each other while more specific interactions were observed between the canonical Sm proteins (Camasses *et al.*, 1998; Fury *et al.*, 1997) (M. Fromont-Racine, A. Brunet-Simon and P. Legrain, unpublished data). Since two-hybrid interactions do not necessarily represent direct protein interactions between the two partners, the observed connections could be mediated by another (Lsm) protein or even by an RNA. Thus, it seems most likely that some of the observed interactions may be mediated by the formation of complexes containing more than two Lsm proteins. In summary, these data support the ability of the Lsm proteins to form a complex or complexes, as also indicated by the finding that Lsm4 can be co-immunoprecipitated with each of the seven other Lsm proteins (Mayes *et al.*, 1999). As both Sm protein and Lsm protein interactions involve the conserved Sm domain, it remains to be determined how these proteins distinguish between each other to form separate complexes.

Lsm proteins are also strongly connected to proteins involved in splicing: SmD2, Prp11, Hsh49, Prp4 and Prp24. A close functional relationship between Prp4 and the U6 snRNP is suggested by genetic studies showing that at non-permissive temperature, mutant *prp4-1* cells exhibit a specific decrease in the level of U6 snRNA (Galisson and Legrain, 1993). Genetic and *in vitro* experiments have led to a model in which Prp24 promotes the annealing/dissociation reactions of U4–U6 dimer during successive rounds of splicing (Ghetti *et al.*, 1995; Raghunathan and Guthrie, 1998; Shannon and Guthrie, 1991). In the U4–U6 snRNP and in the U4–U6–U5 triple snRNP, SmD2 might be part of an interface between the canonical snRNPs and the U6/Lsm particle. In this respect it may be relevant that a two-hybrid screen with the SmE protein selected Lsm3 (SmX4), the paralogue of SmD2, as a prey protein (Camasses *et al.*, 1998). Interactions between the U2 and U6 snRNAs within spliceosomes are well established (Madhani and Guthrie, 1994). The finding of connections between Lsm proteins and U2 snRNA-associated proteins might therefore represent protein interactions at a U2–U6 interface in spliceosomes or during spliceosome formation, as already suggested by the genetic interactions between Prp21 and Prp24, which are U2- and U6-associated, respectively (Vaidya *et al.*, 1996).

More surprisingly, in these genomic screens, we identified a small group of proteins connected to the Lsm proteins and unrelated to pre-mRNA splicing, among them, Psu1, Pat1 and Yel015w. *YEL015W* encodes a protein of unknown function. *PSU1* was initially identified through suppression of the respiratory deficiency of a *pet* mutant (A.A. Tzagoloff, unpublished results), and more recently has been demonstrated to have a role in transcription (Gaudon *et al.*, 1999). Pat1 was previously identified (Rodriguez-Cousino *et al.*, 1995; Wang *et al.*, 1996b) as a topoisomerase II-associated protein. Disruption of the *PAT1* gene causes slow growth and apparently affects the fidelity of chromosome transmission. No role for Pat1 in pre-mRNA splicing has been detected, nor an association with any of the spliceosomal RNAs, although Pat1 co-immunoprecipitates with Lsm proteins (S. Tharun, W. He, A.E. Mayes, P. Lennertz, J.D. Beggs and R. Parker, 2000). This network of interactions revealed an additional strongly connected protein, Xrn1 (Figure 3B). This result suggests an implication of Lsm proteins in the metabolic degradation pathway of mRNAs and is further supported by additional findings: *PAT1* turns out to be equivalent to *MRT1*, in which conditional mutations that inhibit mRNA decapping have been isolated (Hatfield *et al.*, 1996; S. Tharun, W. He, A.E. Mayes, P. Lennertz, J.D. Beggs and R. Parker, 2000). Psu1/Nmd1 was identified as an interacting protein with Upf1, a major player in the non sense-mediated mRNA decay pathway (He and Jacobson, 1995). More significantly, the Psu1 protein has also been implicated in mRNA decapping, and renamed Dcp2 (Dunckley and Parker, 1999). Dcp2 is required for the production of active Dcp1 decapping enzyme, which co-purifies with it. Although it is not a major player in the Lsm screens, Dcp1 was found as prey in a screen with Lsm4 (Table 1, Figure 3B), and Lsm proteins have been found to co-immunoprecipitate along with Dcp1 protein (S. Tharun, W. He, A.E. Mayes, P. Lennertz, J.D. Beggs and R. Parker, 2000). Following the report (Boeck *et al.*, 1998) that Lsm1–Spb8 itself plays a role in mRNA decapping, recent work has shown that mutations affecting several of the Lsm proteins lead to partial inhibition of mRNA decay (S. Tharun, W. He, A.E. Mayes, P. Lennertz,

Copyright © 2000 John Wiley & Sons, Ltd.

J.D. Beggs and R. Parker, 2000). Thus, a network of interactions was found between the Lsm proteins and four proteins that are implicated directly in mRNA turnover: Dcp2/Psu1, Dcp1, Mrt1/Pat1 and Xrn1. Altogether, these data strongly suggest a novel role for an Lsm protein complex in mRNA degradation. This complex could be directly involved in the regulation of mRNA turnover, which is known to be linked to translational initiation (Schwartz and Parker, 1999). Indeed, we found two translational initiation factors, Gcn3 and Gcd11, among the highly specific prey proteins selected by Lsm proteins (Figure 3A). The existence of eight instead of seven Lsm proteins, and the finding of multiple interactions between Lsm proteins and factors involved in mRNA turnover, as well as factors involved in mRNA splicing, raises the possibility that two or more Lsm complexes may exist. Conceivably, alternative Lsm subunit compositions might confer different functional specificity on distinct complexes. In view of these connections, the precise role of Yel015w is currently being investigated.

From the results presented here, it appears that performing multiple genomic screens with functionally related bait proteins and in an iterative manner leads to results whose significance is much greater than the data from the component screens considered separately. Obviously, the frequency with which prey proteins are found also depends on the level of production and stability of the fusion proteins; therefore, while statistical analyses are essential for interpretation of the data, all results should be considered as potentially significant, including single clones, otherwise meaningful interactions may be missed. These questions were also addressed in very recently published studies aiming at a genome-wide description of protein–protein interactions for the yeast proteome (Ito *et al.*, 2000; Uetz *et al.*, 2000) and *Caenorhabditis elegans* (Walhout *et al.*, 2000). However, as opposed to these studies, the strategy used in the present study (see also Flores *et al.*, 1999; Fromont-Racine *et al.*, 1997) aims at the selection of interacting domains instead of checking for interaction between full-length proteins. This leads to a more complete description of the set of interactions and provides in addition information on functional domains. A similar approach has also been successfully performed for the study on the hepatitis C virus proteome (Flajolet *et al.*, 2000). Overall, the results presented here provide a striking illustration that exhaustive and iterative two-hybrid screens can be used on a genome-wide scale to yield functionally meaningful information about protein–protein interactions, and thereby can suggest functions for uncharacterized or partially characterized proteins.

### Acknowledgements

We thank J. Chua, N. Gromak and J. McCormack, who contributed to some of these experiments; C. Marck, for providing DNA Strider; A. Jacquier; and M-L. Ferri, for critically reading the manuscript and F. Lacroute, for the generous gift of Pat1 bait construct and the yeast BMA64 strain. This work was supported in part by the European Union (Biotech 95007—the TAPIR network), by a grant from GIP-HMR and by grant 047685 to JDB from the Wellcome Trust. JDB is supported by a Royal Society Cephalosporin Fund Senior Research Fellowship and AEM was recipient of a Wellcome Trust Prize Studentship.

### References

Achsel T, Brahms H, Kastner B, Bachi A, Wilm M, Lührmann R. 1999. A doughnut-shaped heteromer of human Sm-like proteins binds to the 3′-end of U6 snRNA, thereby facilitating U4/U6 duplex formation *in vitro*. *EMBO J* **18**: 5789–5802.

Allmang C, Petfalski E, Podtelejnikov A, Mann M, Tollervey D, Mitchell P. 1999. The yeast exosome and human PM-Scl are related complexes of 3′ -->5′ exonucleases. *Genes Dev* **13**: 2148–2158.

Banroques J, Abelson JN. 1989. PRP4: a protein of the yeast U4/U6 small nuclear ribonucleoprotein particle. *Mol Cell Biol* **9**: 3710–3719.

Bjorn SP, Soltyk A, Beggs JD, Friesen JD. 1989. PRP4 (RNA4) from *Saccharomyces cerevisiae*: its gene product is associated with the U4/U6 small nuclear ribonucleoprotein particle. *Mol Cell Biol* **9**: 3698–3709.

Boeck R, Lapeyre B, Brown CE, Sach AB. 1998. Capped mRNA degradation intermediates accumulate in the yeast spb8-2 mutant. *Mol Cell Biol* **18**: 5062–5072.

Bordonné R, Tarassov I. 1996. The yeast SME1 gene encodes the homologue of the human E core protein. *Gene* **176**: 111–117.

Branlant C, Krol A, Ebel JP, Lazar E, Haendler B, Jacob M. 1982. U2 RNA shares a structural domain with U1, U4, and U5 RNAs. *EMBO J* **1**: 1259–1265.

Burge CB, Tuschl TH, Sharp PA. 1998. *Splicing of Precursors to mRNA by the Spliceosomes*. Cold Spring Harbor Laboratory Press: New York.

Camasses A, Bragado-Nilsson E, Martin R, Seraphin B, Bordonne R. 1998. Interactions within the yeast Sm core complex: from proteins to amino acids. *Mol Cell Biol* **18**: 1956–1966.

Cooper M, Johnston LH, Beggs JD. 1995. Identification and characterization of Uss1p (Sdb23p): a novel U6 snRNA-

Copyright © 2000 John Wiley & Sons, Ltd. 

associated protein with significant similarity to core proteins of small nuclear ribonucleoproteins. *EMBO J* **14**: 2066–2075.

Dunckley T, Parker R. 1999. The DCP2 protein is required for mRNA decapping in *Saccharomyces cerevisiae* and contains a functional MutT motif. *EMBO J* **18**: 5411–5422.

Erickson FL, Harding LD, Dorris DR, Hannig EM. 1997. Functional analysis of homologs of translation initiation factor 2$\gamma$ in yeast. *Mol Gen Genet* **253**: 711–719.

Fischer U, Sumpter V, Sekine M, Satoh T, Luhrmann R. 1993. Nucleo-cytoplasmic transport of U snRNPs: definition of a nuclear location signal in the Sm core domain that binds a transport receptor independently of the m3G cap. *EMBO J* **12**: 573–583.

Flajolet M, Rotondo G, Daviet L, Bergametti F, Inshauspé G, Tiollais P, Transy C, Legrain P. 2000. A genomic approach of the hepatitis C virus generates a protein interaction map. *Gene* **242**: 369–379.

Flores A, Briand JF, Gadal O, Andrau JC, Rubbi L, Van Mullem V, Boschiero C, Goussot M, Marck C, Carles C, Thuriaux P, Sentenac A, Werner M. 1999. A protein–protein interaction map of yeast RNA polymerase III. *Proc Natl Acad Sci U S A* **96**: 7815–7820.

Fromont-Racine M, Rain JC, Legrain P. 1997. Toward a functional analysis of the yeast genome through exhaustive two-hybrid screens. *Nature Genet* **16**: 277–282.

Fury MG, Zhang W, Christodoulopoulos I, Zieve GW. 1997. Multiple protein: protein interactions between the snRNP common core proteins. *Exp Cell Res* **237**: 63–69.

Fury MG, Zieve GW. 1996. U6 snRNA maturation and stability. *Exp Cell Res* **228**: 160–163.

Galisson F, Legrain P. 1993. The biochemical defects of prp4-1 and prp6-1 yeast splicing mutants reveal that the PRP6 protein is required for the accumulation of the [U4/U6.U5] tri-snRNP. *Nucleic Acids Res* **21**: 1555–1562.

Gaudon C, Chambon P, Losson R. 1999. Role of the essential yeast protein PSU1 in the transcriptional enhancement by the ligand-dependent activation function AF-2 of nuclear receptors. *EMBO J* **18**: 2229–2240.

Ghetti A, Company M, Abelson J. 1995. Specificity of Prp24 binding to RNA: a role for Prp24 in the dynamic interaction of U4 and U6 snRNAs. *RNA* **1**: 132–245.

Gottschalk A, Neubauer G, Banroques J, Mann M, Lührmann R, Fabrizio P. 1999. Identification by mass spectrometry and functional analysis of novel proteins of the yeast [U4/U6.U5] tri-snRNP. *EMBO J* **18**: 4535–4548.

Gottschalk A, Tang J, Puig O, Salgado J, Neubauer G, Colot HV, Mann M, Seraphin B, Rosbash M, Luhrmann R, Fabrizio P. 1998. A comprehensive biochemical and genetic analysis of the yeast U1 snRNP reveals five novel proteins. *RNA* **4**: 374–393.

Hatfield L, Beelman CA, Stevens A, Parker R. 1996. Mutations in trans-acting factors affecting mRNA decapping in *Saccharomyces cerevisiae*. *Mol Cell Biol* **16**: 5830–5838.

He F, Jacobson A. 1995. Identification of a novel component of the nonsense-mediated mRNA decay pathway by use of an interacting protein screen. *Genes Dev* **9**: 437–454.

Hermann H, Fabrizio P, Raker VA, Foulaki K, Hornig H, Brahms H, Luhrmann R. 1995. snRNP Sm proteins share two evolutionarily conserved sequence motifs which are involved in Sm protein–protein interactions. *EMBO J* **14**: 2076–2088.

Ito T, Tashiro K, Muta S, Ozawa R, Chiba T, Nishizawa M, Yamamoto K, Kuhara S, Sakaki Y. 2000. Toward a protein–protein interaction map of the budding yeast: a comprehensive system to examine two-hybrid interactions in all possible combinations between the yeast proteins. *Proc Natl Acad Sci U S A* **97**: 1143–1147.

Jacobs JS, Anderson AR, Parker RP. 1998. The 3′ to 5′ degradation of yeast mRNAs is a general mechanism for mRNA turnover that requires the SKI2 DEVH box protein and 3′ to 5′ exonucleases of the exosome complex. *EMBO J* **17**: 1497–1506.

Jacobson MR, Rhoadhouse M, Pederson T. 1993. U2 small nuclear RNA 3′ end formation is directed by a critical internal structure distinct from the processing site. *Mol Cell Biol* **13**: 1119–1129.

Jandrositz A, Guthrie C. 1995. Evidence for a Prp24 binding site in U6 snRNA and in a putative intermediate in the annealing of U6 and U4 snRNAs. *EMBO J* **14**: 820–832.

Johnson AW. 1997. Rat1p and Xrn1p are functionally interchangeable exoribonucleases that are restricted to and required in the nucleus and cytoplasm, respectively. *Mol Cell Biol* **17**: 6122–6130.

Luhrmann R, Kastner B, Bach M. 1990. Structure of spliceosomal snRNPs and their role in pre-mRNA splicing. *Biochim Biophys Acta* **1087**: 265–292.

Madhani HD, Guthrie C. 1994. Dynamic RNA-RNA interactions in the spliceosome. *Ann Rev Genet* **28**: 1–26.

Mattaj IW. 1986. Cap trimethylation of U snRNA is cytoplasmic and dependent on U snRNP protein binding. *Cell* **46**: 905–911.

Mayes AE, Verdone L, Legrain P, Beggs JD. 1999. Characterisation of Sm-like proteins in yeast and their association with U6 snRNA. *EMBO J* **18**: 4321–4331.

Pannone BK, Xue D, Wolin SL. 1998. A role for the yeast La protein in U6 snRNP assembly: evidence that the La protein is a molecular chaperone for RNA polymerase III transcripts. *EMBO J* **17**: 7442–7453.

Pavitt GD, Ramaiah KV, Kimball SR, Hinnebusch AG. 1998. eIF2 independently binds two distinct eIF2B subcomplexes that catalyze and regulate guanine-nucleotide exchange. *Genes Dev* **12**: 514–526.

Raghunathan PL, Guthrie C. 1998. A spliceosomal recycling factor that reanneals U4 and U6 small nuclear ribonucleoprotein particles. *Science* **279**: 857–860.

Rigaut G, Shevchenko A, Rutz B, Wilm M, Mann M, Séraphin B. 1999. A generic protein purification method for protein complex characterization and proteome exploration. *Nature Biotechnol* **17**: 1030–1032.

Rodriguez-Cousino N, Lill R, Neupert W, Court DA. 1995. Identification and initial characterization of the cytosolic protein Ycr77p. *Yeast* **11**: 581–585.

Roy J, Zheng B, Rymond BC, Woolford JL Jr. 1995. Structurally related but functionally distinct yeast Sm D core small nuclear ribonucleoprotein particle proteins. *Mol Cell Biol* **15**: 445–455.

Rymond BC, Rokeach LA, Hoch SO. 1993. Human snRNP polypeptide D1 promotes pre-mRNA binding in yeast and defines non-essential yeast Smd1p sequences. *Nucleic Acids Res* **21**: 3501–3505.

Salgado-Garrido J, Bragado-Nilsson E, Kandels-Lewis S, Seraphin B. 1999. Sm and Sm-like proteins assemble in two

Copyright © 2000 John Wiley & Sons, Ltd. 

related complexes of deep evolutionary origin. *EMBO J* **18**: 3451–3462.

Sambrook J, Fritsch EF, Maniatis T. 1989. *Molecular Cloning: A Laboratory Manual*, 2nd edn. Cold Spring Harbor Laboratory Press: New York.

Schwartz DC, Parker R. 1999. Mutations in translation initiation factors lead to increased rates of deadenylation and decapping of mRNAs in *Saccharomyces cerevisiae*. *Mol Cell Biol* **19**: 5247–5256.

Seraphin B. 1995. Sm and Sm-like proteins belong to a large family: identification of proteins of the U6 as well as the U1, U2, U4 and U5 snRNPs. *EMBO J* **14**: 2089–2098.

Shannon KW, Guthrie C. 1991. Suppressors of a U4 snRNA mutation define a novel U6 snRNP protein with RNA-binding motifs. *Genes Dev* **5**: 773–785.

Stevens S, Abelson J. 1999. Purification of the yeast U4/U6.U5 small nuclear ribonucleoprotein particle and identification of its proteins. *Proc Natl Acad Sci U S A* **96**: 7226–7231.

Tharun S, He WH, Mayes AE, Lennertz P, Beggs JD, Parker R. 2000. Yeast Sm-like proteins function in MRNA decapping and decay. *Nature* **404**: 515–518.

Uetz P, Giot L, Cagney G, Mansfield TA, Judson RS, Knight JR, Lockshon D, Narayan V, Srinivasan M, Pochart P, Qureshi-Emili A, Li Y, Godwin B, Conover D, Kalbfleisch T, Vijayadamodar G, Yang MJ, Johnston M, Fields S, Rothberg JM. 2000. A comprehensive analysis of protein–protein interactions in *Saccharomyces cerevisiae*. *Nature* **403**: 623–627.

Vaidya VC, Seshadri V, Vijayraghavan U. 1996. An extragenic suppressor of prp24-1 defines genetic interaction between PRP24 and PRP21 gene products of *Saccharomyces cerevisiae*. *Mol Gen Genet* **250**: 267–276.

Vankan P, McGuigan C, Mattaj IW. 1990. Domains of U4 and U6 snRNAs required for snRNP assembly and splicing complementation in *Xenopus* oocytes. *EMBO J* **9**: 3397–3404.

Vojtek AB, Hollenberg SM, Cooper JA. 1993. Mammalian Ras interacts directly with the serine/threonine kinase Raf. *Cell* **74**: 205–214.

Wach A, Brachat A, Pohlmann R, Philippsen P. 1994. New heterologous modules for classical or PCR-based gene disruptions in *Saccharomyces cerevisiae*. *Yeast* **10**: 1793–1808.

Walhout AJM, Sordella R, Lu XW, Hartley JL, Temple GF, Brasch MA, Thierry-Mieg N, Vidal M. 2000. Protein interaction mapping in *Caenorhabditis elegans* using proteins involved in vulval development. *Science* **287**: 116–122.

Wang H, Kohalmi SE, Cutler AJ. 1996a. An improved method for polymerase chain reaction using whole yeast cells. *Anal Biochem* **237**: 145–146.

Wang X, Watt PM, Louis EJ, Borts RH, Hickson ID. 1996b. Pat1: a topoisomerase II-associated protein required for faithful chromosome transmission in *Saccharomyces cerevisiae*. *Nucleic Acids Res* **24**: 4791–4797.

Will C, Lührmann R. 1997. snRNP structure and function, *Eukaryotic mRNA Processing*. IRL Press: Carol Stream, IL; 130–173.

Copyright © 2000 John Wiley & Sons, Ltd. 
